# Determinants of Intravenous Infusion Longevity and Infusion Failure via a Nonlinear Model Analysis of Smart Pump Event Logs: Retrospective Study

**DOI:** 10.2196/48628

**Published:** 2023-09-13

**Authors:** Arash Kia, James Waterson, Norma Bargary, Stuart Rolt, Kevin Burke, Jeremy Robertson, Samuel Garcia, Alessio Benavoli, David Bergström

**Affiliations:** 1 Department of Mathematics & Statistics University of Limerick Limerick Ireland; 2 Medical Affairs Medication Management Solutions, Becton Dickinson Dubai United Arab Emirates; 3 Medical Affairs International Infusion Solutions, Becton Dickinson Winnersh United Kingdom; 4 Systems Engineering International Infusion Solutions, Becton Dickinson Limerick Ireland; 5 Medical Affairs Medication Management Solutions, Becton Dickinson Seville Spain; 6 School of Computer Science and Statistics Trinity College Dublin Dublin Ireland; 7 Research and Development Infusion Acute Care, Becton Dickinson Limerick Ireland

**Keywords:** intravenous infusion, vascular access device, alarm fatigue, intensive care units, intensive care, neonatal, predictive model, smart pump, smart device, health device, infusion, intravenous, nonlinear model, medical device, therapy, prediction model, artificial intelligence, AI, machine learning, predict, predictive, prediction, log data, event log

## Abstract

**Background:**

Infusion failure may have severe consequences for patients receiving critical, short–half-life infusions. Continued interruptions to infusions can lead to subtherapeutic therapy.

**Objective:**

This study aims to identify and rank determinants of the longevity of continuous infusions administered through syringe drivers, using nonlinear predictive models. Additionally, this study aims to evaluate key factors influencing infusion longevity and develop and test a model for predicting the likelihood of achieving successful infusion longevity.

**Methods:**

Data were extracted from the event logs of smart pumps containing information on care profiles, medication types and concentrations, occlusion alarm settings, and the final infusion cessation cause. These data were then used to fit 5 nonlinear models and evaluate the best explanatory model.

**Results:**

Random forest was the best-fit predictor, with an *F*_1_-score of 80.42, compared to 5 other models (mean *F*_1_-score 75.06; range 67.48-79.63). When applied to infusion data in an individual syringe driver data set, the predictor model found that the final medication concentration and medication type were of less significance to infusion longevity compared to the rate and care unit. For low-rate infusions, rates ranging from 2 to 2.8 mL/hr performed best for achieving a balance between infusion longevity and fluid load per infusion, with an occlusion versus no-occlusion ratio of 0.553. Rates between 0.8 and 1.2 mL/hr exhibited the poorest performance with a ratio of 1.604. Higher rates, up to 4 mL/hr, performed better in terms of occlusion versus no-occlusion ratios.

**Conclusions:**

This study provides clinicians with insights into the specific types of infusion that warrant more intense observation or proactive management of intravenous access; additionally, it can offer valuable information regarding the average duration of uninterrupted infusions that can be expected in these care areas. Optimizing rate settings to improve infusion longevity for continuous infusions, achieved through compounding to create customized concentrations for individual patients, may be possible in light of the study’s outcomes. The study also highlights the potential of machine learning nonlinear models in predicting outcomes and life spans of specific therapies delivered via medical devices.

## Introduction

### Overview

Critical care areas require frequent administration of high-alert, critical, short–half-life infusions, intravenous nutrition, sedation and analgesia, as well as other infusions that require rigorous maintenance for continuous delivery. Outside of the intensive care unit (ICU), approximately 60% of all patients will receive an intravenous infusion during their stay [1].

Abrupt and unexpected infusion failure may have severe consequences for patients if the medications are critical, with short–half-life infusions [2]. Continued interruptions to infusions and infusions not running “to time” can also lead to subtherapeutic management. For example, patients receiving antibiotics who require therapeutic drug monitoring based on metrics like area under the concentration-time curve and trough levels often need blood draws before and after administration. The documented time of administration and subsequent blood draws are commonly based on the prescribed regimen and not on the actual completion of the infusion [3,4]. Infusion downstream and upstream occlusion alarms, when substantial, may also contribute to alarm fatigue among clinical staff [5,6]. One extensive study showed that venous access occlusion alarms are responsible for 55% of all intravenous infusion pump alarms in neonatal ICUs [2].

The issue of infusion failure and its determinants has not been comprehensively studied in the literature. Existing in vivo studies have focused on mechanical causes at the vascular access device site [7] and incompatibility issues, either between medications [8] or between medications and administration line materials [9].

A vascular access device (VAD) is defined by the Infusion Nurses Society of the United States as a “catheter, tube, or device inserted into the vascular system, including veins, arteries, and bone marrow” [10]. Definitions for VAD failure include situations where the catheter stops working safely before its intended dwell time or before the traditional 72- to 96-hour dwell time limit [11,12]. Recent guidelines from the Centers for Disease Control and Prevention state that peripheral VADs do not need to be electively resited “more frequently than every 72 to 96 hours” [13]. Using these definitions, the VAD failure rate has been suggested to be as high as 63%, with mean and median values of 46% and 43%, respectively, across studies [14].

The VAD failure rate has a fundamental relationship with the administration method, with gravity administration having a VAD failure rate twice that of even simple rate control infusion devices [15,16]. Modern infusion devices with increased accuracy for the detection of downstream occlusion issues would be expected to reduce the VAD failure rate further. The management of vascular access and infusions also results in a substantial nursing workload. The Therapeutic Intervention Scoring System-28 allocates 3 points to “multiple intravenous medications” (ie, more than 1 medication, “either as single shots or continuously”), 3 points to any “single vasoactive medication,” 4 points in the case of “multiple vasoactive medications, regardless of types and doses,” and 2 points for the care of a “central venous line.” Therefore, continuous infusions of critical, short–half-life intravenous medications via a central VAD could consume 5 to 9 points from a maximum workload of 46 points that can be undertaken by 1 nurse [17], equating to 10%-19% of a critical care nurse’s total activity time. In a study on nursing workload in ICUs with an average length of stay of 7.7 days, it was found that the mean score based on the Therapeutic Intervention Scoring System-28 was 23 (range 14-32 points) and that nursing time constituted the largest economic cost for ICUs [18].

A 2019 study [19] indicated an “excessive nursing workload” across ICUs that was significantly associated with quality of care. Reducing the number of interventions nurses need to undertake to avoid infusion interruption and to increase infusion longevity would be expected to reduce the baseline of nursing workload in intensive care, high-dependency units, and lower-acuity care areas.

In a 2021 study [20] on the impact of infusion alerts and alarms on nursing workflow, alarms and alerts from both intermittent and continuous infusions were analyzed. Alerts, such as those generated by the Dose Error Reduction System due to dose or rate selection by the clinician outside of the defined limits for individual medications, do not interrupt infusions. The study deemed alerts and alarms as “undesirable error states” and described specific conditions that would interrupt infusions, such as flow occlusion and air-in-line alarms.

In our study, we developed working definitions for infusion longevity, and conversely, infusion failure as follows: infusion longevity may be described as the length of time during which a continuous infusion runs without an alarm state causing unexpected and unplanned interruption to the infusion and comes to an end as a planned cessation. Infusion failure may be described as an infusion that does not reach a planned cessation without clinician interventions to manage unexpected and unplanned interruptions.

### Objectives

This study aimed to identify and rank determinants of the longevity of continuous infusions delivered by syringe drivers through the use of nonlinear predictive models to evaluate key factors, and subsequently, develop and test a model for predicting the likelihood of successful infusion longevity; this also involves determining the best predictive model for future use. We expected the analysis to show therapeutic practices and pump management processes that may assist with infusion longevity. Additionally, we aimed to determine which medications are more likely to cause infusion failure and may warrant more intense observation or access management. We also sought to identify critical care units in which infusion failure is more likely to occur and to assess the likelihood of uninterrupted infusion that can be expected in these care areas.

## Methods

### Ethical Considerations

We collected infusion data from smart syringe pumps of type CareFusion/BD Alaris Plus CC from different hospitals in Spain. These data are part of a larger data aggregation for the European region, which is held as a repository as part of the obligation for medical device manufacturers to maintain vigilant postmarket surveillance programs for regulatory and quality purposes.

These data are collected passively as part of the standard function of infusion pumps, capturing all events including alerts, alarms, fault conditions, and programming of all infusions. No patient data are recorded. As these data are retrospective, and therefore, cannot influence clinician decision-making, do not record any direct information related to individual patient therapy, and are detached from any patient or clinician information, there was no requirement for formal ethics approval. The Medication Management Solutions Medical Affairs Department of the pumps’ manufacturer gave clearance for using these data in this study. The question of any conflict of interest was also addressed at this stage. None was found, as the variables studied are universal to “smart” infusion pumps and are not exclusive to the pumps studied.

### Procedure

The data set was obtained from 384 pumps and contained information about various variables. These variables include the profile, indicating the hospital care unit or ward; medication name or type; infusion rate; medication concentration; syringe brand and syringe size; occlusion setting, indicating the pressure threshold at which the pump alarms for an occlusion; and a configured category label for a dependent variable, indicating if the infusion ended by an unexpected and unplanned occlusion or as a planned cessation. Table 1 shows the values for each categorical variable in our data set.

**Table 1:** Values for different categorical variables in “Hospitals infusion data set: Spain.”

Variable	Skewness statistics
Infusions	158,620
Medications	423
Profiles	42
Syringe brands	11
Occlusions	80,764

We used 5 nonlinear models to fit the data and evaluated them with test data to find the best-fit model. These nonlinear models were the following:

- Random forest: a tree-based ensemble learning method that combines multiple decision trees to make predictions. It has been widely used in medical applications due to its ability to handle complex data sets with high performance [21,22].
- XGBoost: a gradient boosting algorithm that uses a series of weak decision trees so that each tree improves the prediction of the previous one. It is known for its speed and ability to handle large data sets [23].
- K-nearest neighbor (KNN): a nonlinear model that makes predictions based on the closest neighbors to the data point. It is often used for classification and regression problems [24].
- Naive Bayes: a probabilistic algorithm that makes predictions based on Bayes’ theorem. It is commonly used for many applications, including medical data sets. The algorithm’s naive assumption is that there is independence among input variables of the model [25].
- Support vector machine (SVM): a kernel-based algorithm that separates data points by finding the best hyperplane that maximizes the margin between classes. It is often used for classification and regression problems [26].

Choosing the best machine learning model to be used in a study among the hundreds of different available models should be based on their characteristics and their previous success in the field. We chose 2 different ensemble models with extreme gradient boosting along with the random forest model. These models differ in use, and they allowed us to combine multiple models to reach a result. These are well-known models that can be used as delegates of ensemble learning methods. We used KNN as a delegate for nonparametric instance-based learning models. SVM was used as the most commonly used kernel-based learning model. Naive Bayes was tested to check a Bayesian learning model with an independence assumption between the predictors. This set of models covered a large area of different learning natures, and the ideal model selection was made based on finding a global optimum. Undertaking a trial-and-error procedure among hundreds of models with infinite parameter selection was beyond the scope of our resources, but selecting a starting set of different models that represented different learning algorithms gave us a diverse and comprehensive starting point.

SVM’s kernel is a radial basis function with the regularization parameter set to 1. XGBoost uses 100 estimators with both the learning rate and maximum depth set to 1. Our random forest uses 100 decision tree estimators, and it uses the Gini index function as its criterion to measure the split quality in each tree. The nearest neighbor (K) was set to 3 for the KNN model. The Naive Bayes model used a Gaussian function. The parameters were tested on a validation set of 20% of the entire data set before running the final output of sample testing.

We then evaluated the performance of each model using an *F*_1_-score and a 5-fold cross validation. *F*_1_-score is a widely used performance metric in classification tasks that measures the balance between precision and recall. It is the harmonic mean of precision and recall, which means that it takes into account both false positives and false negatives, giving equal weight to both [27]. The *F*_1_-score ranges from 0 to 1, where a score of 1 represents perfect precision and recall, and a score of 0 represents poor performance.

The formula for the *F*_1_-score is as follows:

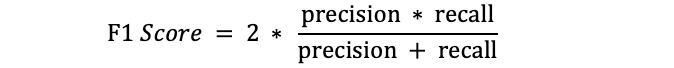



In this formula, precision is the number of true positives divided by the sum of true positives and false positives, and recall is the number of true positives divided by the sum of true positives and false negatives.

The *F*_1_-score was introduced by Van Rijsbergen [28] in 1979 as a way to evaluate the effectiveness of information retrieval systems; since then, it has been widely adopted in various fields, including natural language processing, machine learning, and computer vision. *F*_1_-score is particularly useful when the data set has an imbalance, implying a significant difference in the number of instances for each class; it takes into account both precision and recall, which can be affected by imbalanced data sets.

The target variable was binary, with an imbalance ratio (IR) of 1.05. The IR is defined as the ratio of the majority class to the minority class and is the alternative to skewness in binary classifiers. As a rule of thumb, all IRs less than 1.5 are considered to represent balanced data sets [29,30]. As for the skewness of predictor variables, they can only affect the performance of the models and not the selection of the *F*_1_-score as an evaluation method, as the *F*_1_-score is calculated on the target variable and encompasses both precision and recall. Variables such as profile, medication, and syringe brand are categorical, and variables like infusion rate, concentration dose, occlusion setting, and syringe size are continuous. Therefore, we chose to limit ourselves to calculating only the Pearson skewness ratio statistics for continuous (numerical) variables (Table 2). The formula used to calculate the skewness ratio is as follows:

Skewness ratio = (3(mean(x) - median(x))) ⁄ (standard deviation(x))

The skewness ratios showed that there is no high skewness present in the predictor variables.

**Table 2:** Skewness statistics with imbalance ratios for the numerical data. On the target variable, the data set had an imbalance ratio of 1.05.

Variable	Skewness statistics		
	Mean	Median	Skewness ratio
Infusion rate	6.28	2.0	1.05
Concentration dose	13.11	2.0	1.22
Syringe size	49.67	50	–0.27
Occlusion setting	175.86	200	–0.27

In this study, we set a default threshold of 0.5 to transform predicted probabilities into binary class labels. This approach, commonly used in similar studies, balances precision and recall. Although not a parameter within the models, this threshold selection is an essential postprocessing step that substantially influences categorizing instances as positive or negative.

The efficacy of our selected 0.5 thresholds is substantiated by the balanced precision and recall rates observed in our results. This aligns effectively with our research objectives. We understand that different applications may require different thresholds, but we suggest that our choice of 0.5 is appropriate due to its consistent performance across various models and data sets. As part of our future endeavors, we are keen to investigate dynamic threshold selections. We recognize that this could significantly influence our study’s outcomes.

The best-performing model was chosen as the final analysis model. We also calculated the *F*_1_-score for a model that consistently resulted in the majority class in the data set, which is the occlusion class in our data set. We called this model the “majority voting model.”

In the Results section the selection of random forest as the best-fit model for our data is explained. These results derive from the 5-fold cross-validation technique, where we divided the data set into 5 equal subsets. With each test, 1 subset was run as the test set while we attempted to fit our model with the other 4 subsets as the training set. The 5-fold cross-validation technique is a good modelling practice because it helps to mitigate the problem of overfitting and provides a more accurate estimation of model performance. It is a commonly used approach because it balances the trade-off between the number of folds and the variance in the estimated performance metrics [25].

Once we identified random forest as the best-fit model, we used it to calculate each variable’s importance to infusion longevity and to identify the most important predictors of unexpected infusion failure. Variable importance measures the contribution of each variable to the model’s overall fitness power. Random forest is a popular machine learning algorithm that combines multiple decision trees to make more accurate predictions. One important aspect of random forest is the calculation of feature importance, which helps to identify which features have the most impact on the prediction.

Feature importance is calculated by analyzing the contribution of each variable in the decision-making process of each individual tree within the random forest model. The importance of a feature is determined by calculating the total reduction of the impurity measure achieved by splitting on that variable across all trees in the forest [21]. In other words, variables that are able to create the largest reduction in impurity (eg, Gini index or entropy) are considered the most important variables.

The importance scores of each variable are then normalized to ensure that they add up to 1, so they can be compared to one another. This enables researchers to identify which features are most relevant for predicting the target variable or model fitness.

In summary, variable importance in random forest is calculated by measuring the impact of each variable in the decision-making process of each individual tree and then aggregating these values across all trees in the forest. The resulting scores can help researchers to identify the most important features for predicting the target variable [31].

## Results

As noted above, random forest was the best-fit model for the data set (Table 3).

**Figure 1:**
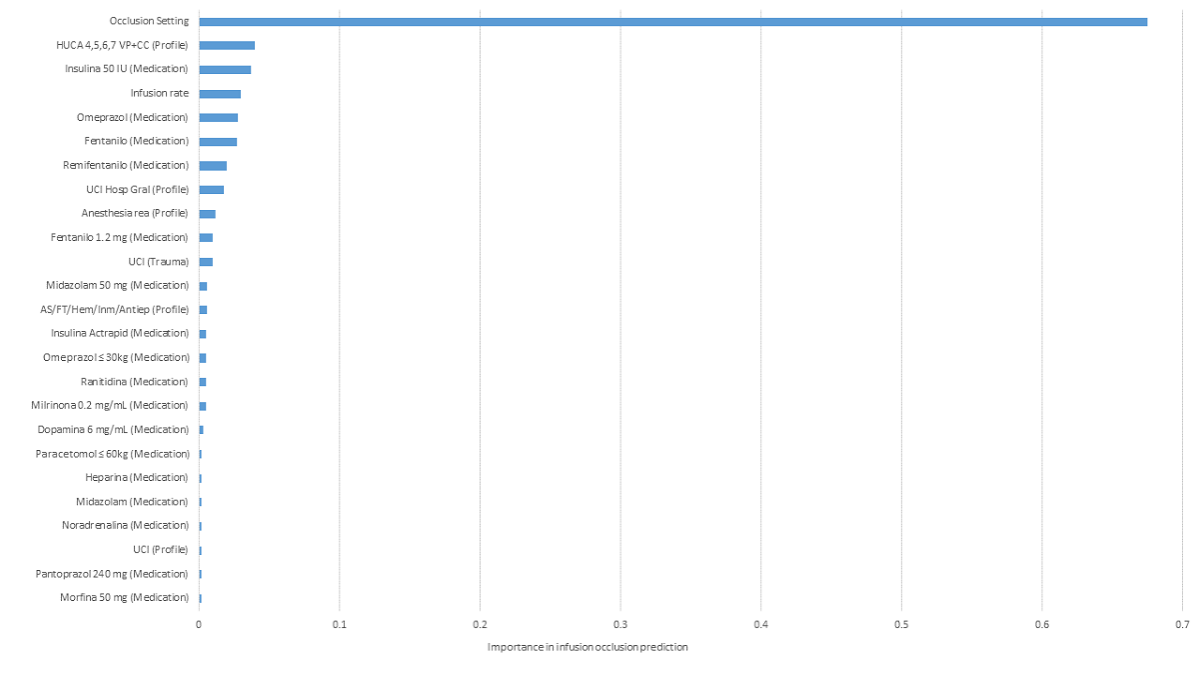
Variable importance in infusion occlusion prediction for “Hospitals infusion data set: Spain” for CareFusion/BD Alaris Plus syringe pumps.

As random forest outperformed all other models and had the highest *F*_1_-score, it was selected to predict infusion occlusion in smart syringe infusion pumps of type CC in “Hospitals infusion data set: Spain.” The results are provided in Figure 1.

**Table 3:** The *F*_1_-score of all the selected models’ fits to the infusion data set. The results show that random forest was the best-fit model for our data.

Model	*F*_1_-score
Majority voting model	67.48
Extreme gradient boosting	79.63
Random forest	80.42
Support vector machine	77.42
Naive Bayes	75.04
K-nearest neighbor	75.73

**Figure 2:**
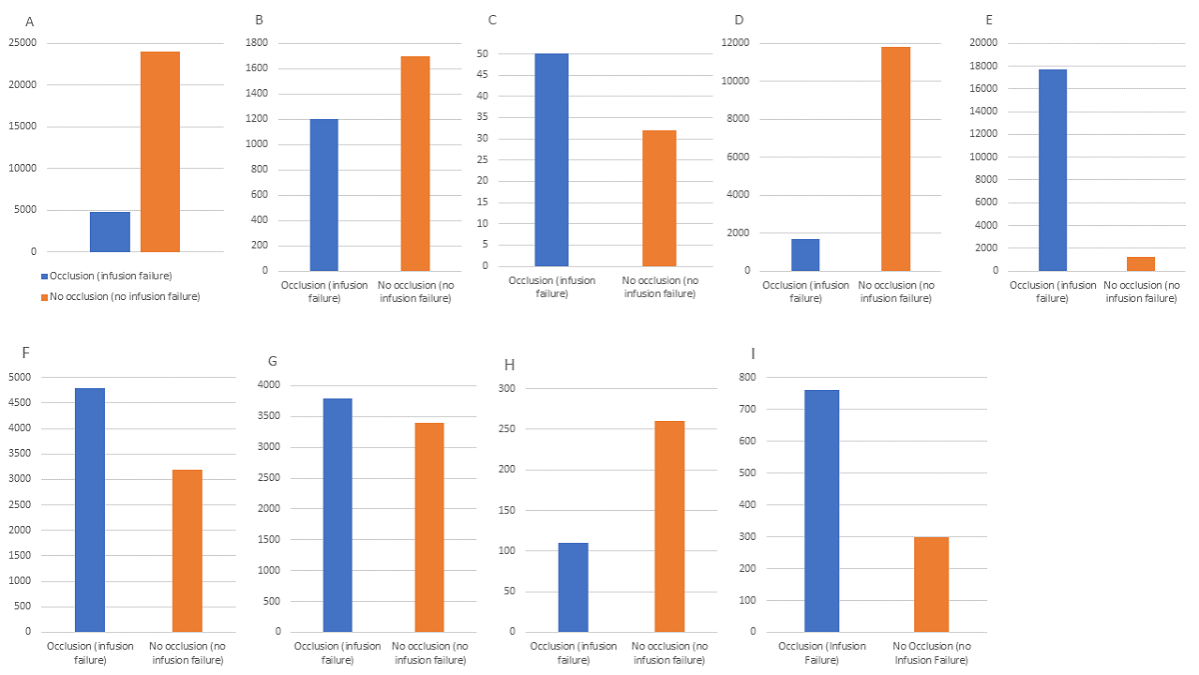
Total number of infusions with and without occlusion for binary variables, which are essentially bound to the treatment process or location and beyond the direct control or manipulation of clinicians. (A) HUCA 4.5 6.7 VP+CC (profile). (B) Insulin 1 IU/mLl (medication). (C) Omeprazole (medication). (D) Fentanil (medication). (E). UCI Hosp Gral (profile). (F) Anesthesia Rea (profile). (G) Fentanil 1.2 Mg (medication). (H) UCI trauma (profile). (I) Remifentanil (medication).

Figure 2A-I shows the total number of infusions with and without occlusions for binary variables, which are essentially bound to the treatment process or location and are beyond the direct control or manipulation of clinicians. The application of profiles differs widely among end-user facilities dependent on their structure, risk strategy, and the services they provide. The nomenclature is “free text” and is also language dependent. For example, the term “anesthesia rea” seen here would usually pertain to resuscitation area usage and the operating room in several European languages. The profile “HUCA 4.5 6.7 VP+CC” may mean that the hospital has a mix of different pump types from different manufacturers as particular pumps are mentioned in the profile title. Generally, profiles are given care units, such as neonatal intensive care, adult intensive care, pediatric oncology, and labor and delivery. Therefore, although there is a strong degree of harmonization across facilities and patient types within profiles, as with all multicenter data, there may be differences in acuity; an ICU in a university-level facility will likely have far higher patient acuity levels than a general tertiary care unit. This said, general patient characteristics by profile, in terms of weight, medication concentrations used, and other infusion parameters, may reasonably be expected to be uniform across profiles pertaining to each discipline [2].

**Figure 3:**
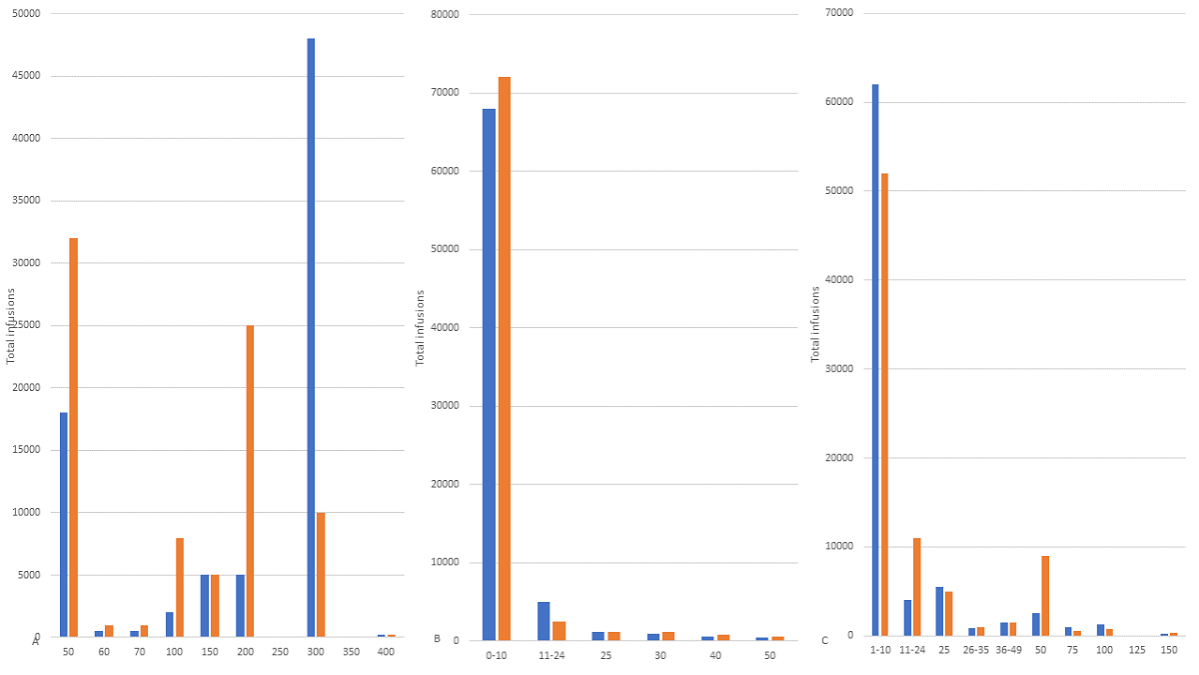
Occlusion versus no occlusion in nonbinary variables of (A) occlusion threshold (mm Hg), (B) infusion rate (mL/hr), and (C) concentration (units/mL). these variables are within the control of clinicians or clinical teams. Concentration units pertain to several units in the International System of Units per ml (eg, mg, mcg, ng, and IU). Blue indicates no occlusion (no infusion failure) and orange indicates occlusion (infusion failure).

Figure 3 illustrates the total number of infusions with and without occlusions across varying values of important nonbinary variables, which are within the control of clinicians or clinical teams.

**Figure 4:**
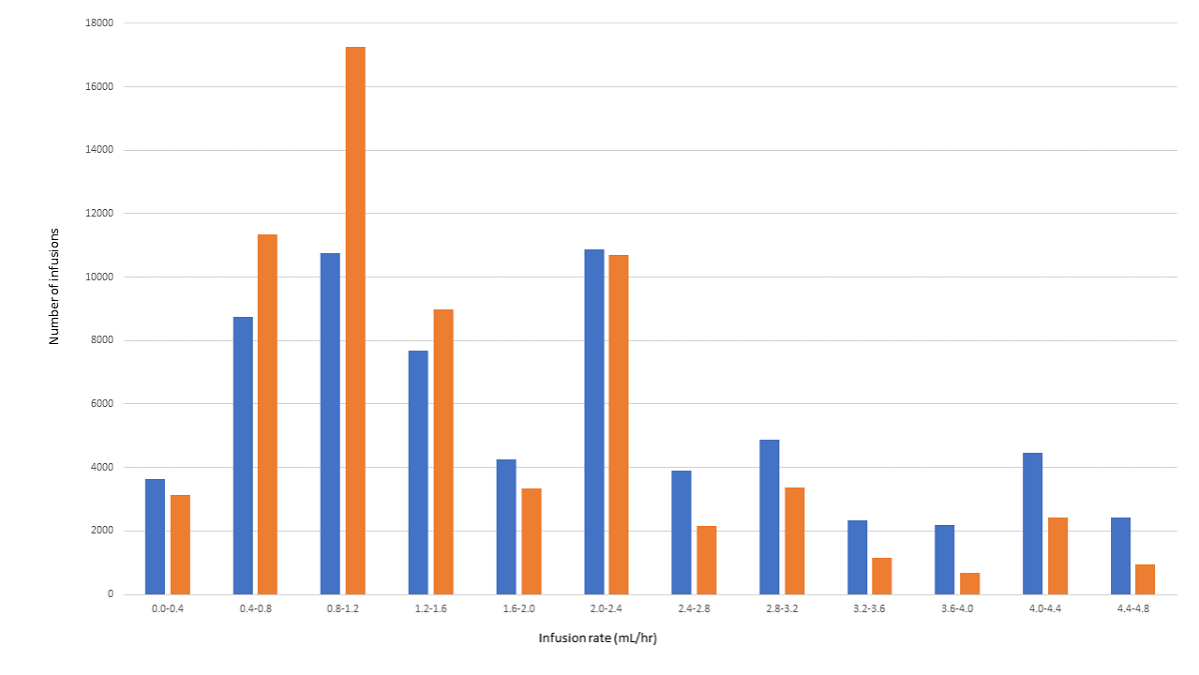
Low-flow infusion rates versus the number of infusions with occlusion (unexpected infusion interruption) and no occlusion (planned infusion cessation). Blue indicates no occlusion and orange indicates occlusion.

Figure 4 shows a more detailed breakdown of continuous low-rate infusions by rate. These low-rate infusions are of particular interest and importance clinically, as they are commonly critical short–half-life medication infusions, which are titrated to effect, and their low-rate infusions can cause a longer time to alarm, leading to reduced detectability of “no delivery” states.

## Discussion

### Study Limitations

Data were only collected from hospitals in Spain, though the investigational method could be applied to other regions as the software deployed in the smart pumps is available worldwide and the structure and deployment of the medication library from which the data were gathered has been found to be very similar in a previous wide-ranging study of event logs [2].

The study was limited to one type of syringe pump, the CareFusion/BD Alaris CC pump. Similarly, the investigational model can be applied to other pumps, as features like profile, syringe types and brands, medication libraries, and occlusion alarm pressure settings are considered to be universal across syringe pumps. However, one caveat is that the devices in this study have a fairly unique feature, that of in-line pressure monitoring, where the vein pressure of the patient at the point of the VAD is transmitted directly to a dedicated pressure sensor situated downstream of the syringe and in direct line connection to the VAD. The occlusion alarm setting in this device can be set as low as 15 mm Hg above the detected vein pressure, though commonly, this is set at 30 mm Hg above the vein pressure in neonatal care, and some units use an “auto-offset” feature for automatically setting the alarm after 15 minutes of infusion [32]. Generally, syringe driver infusion pumps without in-line pressure monitoring transmit these data from the drive head behind the syringe, which involves added variables of medication viscosity, syringe friction, and a minimum level of pressure detection, which is rarely below 85 mm Hg.

The question of the type of vascular access of each patient in the study was also a limitation, and whether the VAD was central, peripheral, or umbilical in the case of neonates or a “long line,” such as peripherally inserted central catheters, could not be answered given the data available. However, some inferences could be made from clinically acceptable maximum concentrations for peripheral administration from facility protocols. That being said, evidence is accruing that the concentration of administered individual medications (concentration was not identified as a substantial determinant in our study) is not per se as important as the contact time between medications when multiple infusions pass through a single VAD, along with the subsequent interactions between them. [7,8] At low-flow rates, this contact time is extended, with more time for reactions between medications and subsequent precipitate production. In-line filtration to protect the VAD from precipitate occlusion is emerging from the available evidence as an important factor in determining VAD longevity beyond that of VAD type and medication concentration [7,8].

Including more variables to the information directly available from smart pumps in the analysis, such as direct information about the VAD type and other infusions running via one VAD, may provide a more comprehensive understanding of the in vivo factors that influence infusion longevity.

### Principal Findings

The occlusion alarm setting threshold was the most important variable for infusion longevity, and beyond 2 individual medications, the infusion rate was the next most important variable. The data drill-down (Figure 3) and ratio ranking (Tables 4 and 5) show that lower ratios of occlusion to no occlusion were associated with higher rates of infusion, with the rate bracket of >3.6-≤4.0 mL/hr having the best ratio at 0.311. However, the bulk of infusions in the study run at rates far lower than that, with 60.11% running at below 2.0 mL/hr. This is understandable given the fluid balance (or more correctly, the fluid restriction requirements) of medication infusions in critical care, particularly in neonatology and pediatrics. Due to maintaining critical care patients’ nutrition as well as managing renal failure and fluid balance, it is not uncommon to use higher concentration infusions to deliver continuous infusion doses with smaller volumes.

Considering the findings of this study and in vitro studies of infusion startup delay and infusion “no-flow” interruptions [33], as well as the influence of administration line compliance [34], filters [35], the interplay between multiple infusions [36], and resistance from backcheck and antisyphon valves [35], the risks of protracted and clinically important nondelivery and occlusion are likely at low rates, particularly below rates of 0.5 mL/hr [35]. The study’s findings suggest a balance between the need to restrict fluid delivery to patients and maintaining the integrity and longevity of infusion might be best achieved with a rate ranging from >2.0 to ≤2.4 mL/hr (ratio 0.985), although the next higher rate of >2.4-≤2.8 mL/hr would yield a far better ratio of 0.553, albeit with some compromise in fluid restriction control.

**Table 4:** Ratios of occlusion versus no-occlusion infusions at investigated flow rates (N=131,654).

Rate (mL/hr)	0.0-≤0.4	>0.4-≤08	>0.8-≤1.2	>1.2-≤1.6	>1.6-≤2.0	>2.0-≤2.4	>2.4-≤2.8	>2.8-≤3.2	>3.2-≤3.6	>3.6-≤4.0	>4.0-≤4.4	>4.4-≤4.8
Ratio (occlusion vs no occlusion)	0.859	1.297	1.604	1.169	0.786	0.985	0.553	0.691	0.488	0.311	0.547	0.387
Occlusion, n (%)	3635 (53.8)	8741 (43.5)	10,759 (38.4)	7686 (46.1)	4266 (56.0)	10,868 (50.4)	3909 (64.4)	4887 (59.1)	2340 (67.2)	2189 (76.3)	4452 (64.6)	2423 (72.1)
No occlusion, n (%)	3121 (46.2)	11,338 (56.5)	17,261 (61.6)	8986 (53.9)	3352 (44.0)	10,708 (49.6)	2160 (35.6)	3376 (40.9)	1142 (32.8)	680 (23.7)	2437 (35.4)	938 (27.9)
Total infusions studied, n (%)	6756 (5.13)	20,079 (15.25)	28,020 (21.28)	16,672 (12.66)	7618 (5.79)	21,576 (16.39)	6069 (4.61)	8263 (6.28)	3482 (2.64)	2869 (2.18)	6889 (5.23)	3361 (2.55)

**Table 5:** Ratios of occlusion versus no-occlusion infusions, ranked by the best-performing rate according to the ratio (N=131,654). The possible optimal rate ranges are 2.4-2.8 mL/hr (0.553) and 2.0-2.4 mL/hr (0.985).

Ranking by ratio	1	2	3	4	5	6	7	8	9	10	11	12
Rate (mL/hr)	>3.6-≤4.0	>4.4-≤4.8	>3.2-≤3.6	>4.0-≤4.4	>2.4-≤2.8	>2.8-≤3.2	>1.6-≤2.0	0.0-≤0.4	>2.0-≤2.4	>1.2-≤1.6	>0.4-≤08	>0.8-≤1.2
Ratio (occlusion vs no occlusion)	0.311	0.387	0.488	0.547	0.553	0.691	0.786	0.859	0.985	1.169	1.297	1.604
Total infusions studied, n (%)	2869 (2.18)	3361 (2.55)	3482 (2.64)	6889 (5.23)	6069 (4.61)	8263 (6.28)	7618 (5.79)	6756 (5.13)	21,576 (16.39)	16,672 (12.66)	20,079 (15.25)	28,020 (21.28)

Therefore, we suggest that, when feasible, some relaxation of fluid restriction and medication concentrations be considered to deliver infusions at rates between 2.0 and 2.8 mL/hr and higher, if at all possible, for continuous infusions. The improvement in the occlusion ratio may be related to the simple volume of medication moving through the VAD and “flushing” it more effectively than very low-rate infusions can achieve, or it could be attributed to the previously mentioned concept of reduced contact time between medications being administered through a single VAD at higher rates. It is possible to target this flow rate range even through wider titration ranges by manipulation of the final concentration of medications. The suggested rate range would also assist with the clinical detectability of nondelivery [33,35].

In Figure 2A-I, binary variables linked to the treatment process or unit type are identified. These variables are essentially beyond the direct control or manipulation of clinicians. However, this information remains valuable as a “high-risk” indicator for individual medications that may benefit from concentration manipulation to facilitate higher delivery rates, closer observation of the infusion, or central VAD delivery and exclusive-line administration rather than peripheral administration along with multiple infusions. A multidisciplinary approach to the management of such high-risk medications is advocated.

### Conclusions

These findings have important implications for health care professionals who use smart infusion pumps to deliver medications to patients. The study may assist health care professionals to make informed decisions regarding the medication to be administered, concentrations to be used, and infusion duration or rate, to improve infusion longevity, reduce the risk of unplanned infusion interruption, and mitigate risks to the VAD.

The study also highlights the potential of machine learning nonlinear models to predict infusion occlusions in smart infusion pumps. The process of selecting the most appropriate model could be applied to studies involving other medical devices.

## Abbreviations

ICU: intensive care unit
IR: imbalance ratio
KNN: k-nearest neighbor
SVM: support vector machine
VAD: vascular access device
